# Exploring the aftermath of hematopoietic cell transplantation: 18-year insights into post-transplant neoplasms

**DOI:** 10.3389/fonc.2025.1589755

**Published:** 2025-07-22

**Authors:** Rawad Rihani, Shrouq Amer, Khalid Halahleh, Hasan Hashem, Zaid Abdel Rahman, Laith Baqain, Mayada Abu Shanap, Iyad Sultan, Amr Qudeimat

**Affiliations:** ^1^ Department of Pediatrics, Blood and Marrow Transplantation and Cellular Therapy, King Hussein Cancer Center, Amman, Jordan; ^2^ Adult Blood and Marrow Transplantation and Cellular Therapy Program, King Hussein Cancer Center, Amman, Jordan; ^3^ Faculty of Medicine, University of Jordan, Amman, Jordan; ^4^ Department of Bone Marrow Transplantation and Cellular Therapy, St. Jude Children’s Research Hospital, Memphis, TN, United States

**Keywords:** hematopoietic cell transplantation (HCT), second cancer, hematologic malignancies, cancer survivors, human leukocyte antigen (HLA), myeloablative conditioning (MAC), reduced-intensity conditioning (RIC), total body irradiation (TBI)

## Abstract

**Introduction:**

Survival post-hematopoietic stem cell transplantation (HCT) is improving, with an increasing number of survivors. Subsequent neoplasms (SNs) following HCTs are of particular concern.

**Methods:**

Between January 2003 and December 2022, HCT recipients’ records were retrospectively reviewed.

**Results:**

At a median follow-up of 108 months (range, 0.13-215), 2659 patients received HCTs. Of those, 1131 (43%) were <18 years old. Allogeneic HCTs were conducted in 1476 (56%) patients. Myeloablative conditioning (MAC) was utilized in 2157 (81%), and 583(22%) received TBI. At a median of 9 years following transplant, forty-three patients developed SNs (1.6%) with a median age at time of HCT of 27.6 years (range, 2.8-64.8). Of those: 32 were males (74%), 20 received full HLA-matched allogeneic HCTs (46.5%), two (4.6%) had unrelated cord blood HCT (UCB), and one (2.3%) received haplo-HCT, whereas autologous HCTs accounted for 46.6% (n=20). Underlying diseases were: ALL(13.9%), AML(11.6%), Hodgkin Lymphoma(13.9%), Non-Hodgkin lymphoma(13.9%), Multiple Myeloma(18.6%), Fanconi Anemia(6.9%), CML(6.9%),Neuroblastoma(2.3%), and thalassemia (2.3%).); cGVHD occurred in (74%), and CMV infection/reactivation in (60.5%). Stem cell source included peripheral blood in (81.4%), BM in (3.9%), and UCB in (4.7%). Conditioning regimens were MAC (81.4%) vs RIC (18.6%). TBI-based regimen was utilized in 14 patients (32.5%). Subsequent hematologic malignancies accounted for 32.5% of SNs. While subsequent solid neoplasms occurred in 65.2%, and PTLD occurred in 2.3%. The probability of 5-year overall survival after a SN was 58.2%.

**Conclusions:**

SNs adversely impact the overall survival and quality of life of HCT survivors. In our cohort, the rate of post-HCT SNs was lower than that in the literature; however, longer follow-up of our cohort is needed.

## Introduction

Hematopoietic cell transplantation (HCT) is a curative modality for many malignancies and nonmalignant disorders. Significant advances in HCT have been attained. Consequently, more HCT recipients are becoming long-term survivors (1–3). As survivors age, their risk of experiencing post-HCT late effects increases.

HCT survivors face unique long-term challenges after their primary disease has been cured (3). In this setting, subsequent neoplasms (SNs) are among the biggest challenges that can develop after autologous or allogeneic HCT. Although rare, the impact of a SN is concerning due to its high mortality rates (4, 5). In fact, SNs account for 12%-27% of deaths among long-term HCT survivors (6, 7).

The relative risk associated with SNs in HCT recipients is influenced by patient- and treatment-related factors, with estimates ranging from a 2- to 10-fold increased risk (8). Cumulative incidence (CI) varies with follow-up duration: at 10 years, CIs range 2.2%-6.4% (9, 10); at 15 years, 10%-12% (11); and at 20 years, 6.9%-8.6% (12). The CI of a subsequent solid neoplasm (SSN) developing does not appear to plateau and was 3.8% (95% CI, 2.2-5.4) at 20 years post-HCT (12). Moreover, the CI of SNs at 5, 10, and 15 years post-HCT in survivors who received total body irradiation (TBI) conditioning was 0.7%, 2.2%, and 6.7%, respectively, compared to that of SNs at the same time points among those who did not receive TBI, which was 0.3%, 0.6%, and 0.8%, respectively (13). The risk of SNs becomes apparent 5 years post-HCT (4), and the incidence rises over time; several studies with 20-year follow-up have not shown any plateau in SN occurrence (3, 4, 14, 15).

SNs can be categorized into post-HCT Epstein-Barr virus–related B-cell lymphoproliferative diseases (PTLD), acute myeloid leukemia/myelodysplastic syndrome (AML/MDS) after autologous HCT for lymphoma, and SSNs (3, 16–18). PTLD and AML/MDS develop early post-HCT, but SSNs have a longer latency (19). Complex interplay among host, tumor, and environmental characteristics causes SNs to develop (20, 21). These include younger age at HCT (22), higher TBI dose (22–24), genetics (19), graft T-cell depletion, anti–thymocyte globulin use, human leukocyte antigen (HLA)-mismatched donors, chronic graft-versus-host disease (cGVHD), primary disease, and high-intensity chemotherapy and radiotherapy in primary treatment and conditioning regimens (25–27). Furthermore, an immunocompromised state post-HCT and viral infection are associated with increased risk (16, 28). Similarly, cyclophosphamide use is a risk factor for SNs, independent of TBI (29).

Outcomes of patients with SNs have been described by several groups (3, 6, 12, 30). The 5-year overall survival (OS) is 42%-44% after aSN diagnosis (12, 30). In one report, the median OS was 15.7 months for patients with SNs that developed after reduced-intensity conditioning (RIC); OS was 66.4%, 53.3%, and 30.6% at 6, 12, and 60 months, respectively (9).

Here we reviewed the incidence, risk factors, and potential outcomes of SNs among pediatric and adult recipients of HCTs at King Hussein Cancer Center (KHCC) during an 18-year period.

## Methods

### Patient population and data source

Data from 2659 pediatric and adult HCT recipients at KHCC (January 2003–December 2022) were retrospectively reviewed and extracted from the KHCC-HCT Program database and patients’ electronic medical records. The study was conducted in compliance with the KHCC Institutional Review Board’s approval (approval number: [17 KHCC 63], dated [10-May-2017]).

Variables included age at HCT, stem cell source [i.e., bone marrow, peripheral blood stem cells (PBSCs), or umbilical cord blood (UCB)], donor type (related or unrelated), and conditioning regimen intensity. TBI use in conditioning regimens included no TBI, 200 cGy, or 1200 cGy. The timing of HCT was grouped into two eras: 2003–2010 vs 2010–2022. HCT indications were grouped into malignant disorders [i.e., AML, acute lymphoblastic leukemia (ALL), chronic myeloid leukemia (CML), MDS, lymphomas, and solid tumors] and nonmalignant disorders. SN documentation included neoplasm type and treatment received (i.e., chemotherapy and/or radiotherapy and palliation). Age at SN diagnosis, time from HCT to SN diagnosis, and presence of active GVHD at the time of SN diagnosis were also captured.

### Conditioning regimens and GVHD prophylaxis

Conditioning regimen intensity was classified as myeloablative conditioning (MAC) or RIC based on criteria from the Center for International Blood and Marrow Transplant Research (31). GVHD prophylaxis consisted of calcineurin inhibitors combined with methotrexate, mycophenolate mofetil, and/or alemtuzumab, or anti–thymocyte globulin. A combination of methotrexate and prednisone was also used in some patients.

### Post-HCT follow-up, evaluations, and screening:

After their HCT, patients were monitored at KHCC and late effects clinics; they were followed indefinitely, per international guidelines (32, 33). All SNs were confirmed by biopsies, and biopsy specimens were examined by an experienced pathologist at KHCC. Patients whose primary disease was acute or chronic leukemia or AML/MDS had to have a different immunophenotype, FAB subtype, or conventional karyotype for the new tumor to be classified a SN.

### Statistical analyses

Descriptive statistics were used to summarize patient and HCT characteristics at baseline. The main outcome variables of interest were the CI of SNs and OS. CIs of SNs were calculated using the Kaplan-Meier method at various time points post-HCT, with corresponding 95% CIs. The incidence of SNs was determined by dividing the total number of events by the total number of patient-years.

Chi-square statistics were used to assess significant differences in the distribution of parameters between patients who experienced SNs and those who did not. Time-to-event analysis was conducted to determine survival time from the date of HCT or SN diagnosis. OS probabilities and median survival times were estimated using the Kaplan-Meier estimator. The CI of death due to SNs, considering death by all causes, was also calculated.

Cox proportional-hazards models were used to evaluate the impact of various factors on OS. Univariate and multivariate Cox-regression analyses were performed to measure the association between potential risk factors and SN development, and the influence of SNs on OS. Hazard ratios with corresponding 95% CIs were calculated to assess the strength of these associations. Covariates included in multivariate analyses were selected based on their significance in univariate analyses and their clinical relevance. The following variables were examined as potential risk factors for SNs developing after HCT: age at HCT, sex, initial diagnosis, disease duration, latency between HCT and SN, prior radiotherapy, conditioning regimen intensity, use of TBI-based conditioning regimens compared to non-TBI regimens, stem cell source, and donor type. Gray’s test was used to compare the cumulative incidence of SNs between subgroups, including age and gender. A p-value of less than 0.05 was considered statistically significant.

## Result

### Patient characteristics

At median follow-up of 108 months (range, 0.13–215 months), 2659 patients were retrospectively reviewed: 1131 (43%) were younger than 18 years at the time of their HCT; median age at the time of HCT was 27.6 years (range, 2.8-64.8). In the entire cohort, 1604 (60%) were males, and 2087 (78%) were Jordanians. Malignant diseases accounted for 2097 (79%) indications for HCT (**Table 1**). Of the 2097 patients who had a primary malignant disease, 1883 (90%) received HCT for hematologic malignancies: 310 (14.8%), for ALL; 350 (16.7%) for AML; 50 (2.4%) for CML; 102 (4.9%) for MDS; 386 (18.3%) for plasma cell disorders of which the majority were due to multiple myeloma (363/386 (17.3%)); and 685 (32.7%) for lymphoma, the majority of which were for Hodgkin’s lymphoma (453/685 (21.6%)). Solid tumors accounted for 214 (10.2%) of the malignant indications for HCT, whereas the nonmalignant conditions accounted for 562 (21%) (**Table 1**).

**Table 1:** Characteristics of pediatric and adult recipients of HCT and features of their procedures.

Characteristics	Adult patients *n* (%)	Pediatric patients *n* (%)	Entire cohort N (%)
No. of patients	1528 (57%)	1131 (43%)	2659 (100)
Nationality			
* Jordanian*	1263	824	2087 (78)
* Not Jordanian*	265	307	572 (22)
Sex			
* Male*	952	652	1604 (60)
* Female*	576	479	1055 (40)
Primary Diagnoses			
Malignancies	1456	641	2097 (79)
Leukemias	457	355	812 (39)
* ALL*	122	188	310 (38)
* AML*	229	121	350 (43)
* MDS*	70	32	102 (13)
* CML*	36	14	50 (6)
Lymphoma	579	106	685 (33)
Non-Hodgkin’s lymphoma	209	21	230 (33)
Hodgkin’s Lymphoma	368	85	453 (66)
Natural killer/T-cell lymphoma	2	0	2 (1)
Plasma cell disorders	386	0	386 (18)
Multiple Myeloma	363	0	363 (94)
Plasmacytoma	19	0	19 (5)
Plasma cell leukemia	2	0	2 (0.5)
Poems syndrome	2	0	2 (0.5)
Solid tumors	34	180	214 (10)
* Neuroblastoma*	1	121	122 (57)
* Wilms tumor*	0	13	13 (6)
* Sarcoma*	3	10	13 (6)
* Brain tumor*	0	30	30 (14)
* Germ cell tumor*	27	3	30 (14)
* Renal cell carcinoma*	3	0	3 (1)
* Rhabdomyosarcoma*	0	3	3 (1)
Nonmalignant conditions	72	490	562 (21)
Hemoglobinopathies	0	224	224 (40)
Bone marrow failure syndrome	72	144	216 (38)
Immune deficiency	0	96	96 (17)
Metabolic disorders	0	26	26 (5)
HCT Features			
Type of transplant			
* Allogeneic stem cells*	606	870	1476 (56)
* Autologous stem cells*	921	262	1183 (44)
Conditioning regimen			
* MAC*	1312	845	2157 (81)
* RIC*	214	259	473 (18)
* No conditioning*	2	27	29 (1)
Total body irradiation			
* Yes*	256	327	583 (22)
* No*	1272	804	2076 (78)
Allogeneic donor type			
Full HLA-matched related donor	526	676	1202 (81)
* MS donor*	504	579	1083 (90)
* Fully matched family member*	22	97	119 (10)
Alternate donor	80	194	274 (19)
* Haploidentical donor*	73	156	229 (84)
* Unrelated UCB*	3	37	40 (14)
* Matched unrelated donor*	4	1	5 (2)
Stem cell source			
* Bone marrow*	80	250	330 (12)
* PBSCs*	1445	839	2284 (86)
* UCB*	3	42	45 (2)

ALL, acute lymphoblastic leukemia; AML, acute myeloid leukemia; CML, chronic myeloid leukemia; HCT, hematopoietic cell transplantation; HLA, human leukocyte antigen; MAC, myeloablative conditioning; MDS, myelodysplastic syndrome; MS, matched sibling; No., number; PBSC, peripheral blood stem cells; RIC, reduced-intensity conditioning; UBC, umbilical cord blood.

### Hematopoietic cell transplantation features and outcomes

Allogeneic HCTs were conducted in 1476 (56%) patients; median time from diagnosis to HCT was 1.3 months (range, 0.4–101 months). MAC conditioning was utilized in 2157 (81%) cases, and TBI-based conditioning was administered in 583 (22%) cases. Leukemia (812, 39% of malignant conditions) was the most common indication for allogeneic HCT. Full HLA-matched related donors were used in 1202 (81%) HCTs, and alternate donors were used in 274 (19%) HCTs: 229 (84%) involved a haploidentical donor, 40 (14%) used UCB and 5 (2%) matched unrelated donors. PBSCs were the predominant source (2284, 86%), followed by bone marrow (330, 12%) and UCB (45, 2%) (**Table 1**). CGVHD was reported in 243 (16%) patients who underwent Allogeneic HCTs.

Among the 2,659 patients included in the study, a total of 1,166 patients (43.8%) experienced either persistent or relapsed disease following HCT. Of the entire cohort, 222 patients (8.3%) underwent more than one transplant, either due to disease relapse or graft failure. Among the 43 patients who developed SNs, only 4 had undergone more than one transplant: 3 patients with multiple myeloma and 1 patient with Fanconi anemia. The remaining majority received a single transplant as part of their treatment course.

Median OS of the entire cohort was 115 months (95% CI, 13.7–216 months); the 5-year OS was 89.3% (95% CI, 87.9-90.8), and the 10-year OS was 75% (95% CI, 73-77). There was no difference in the risks of SN developing related to the type of HCT (allogeneic *vs* autologous), intensity of conditioning regimens (RIC *vs* MAC), or alkylators use.

### Risk of subsequent neoplasms

**Figure 1:**
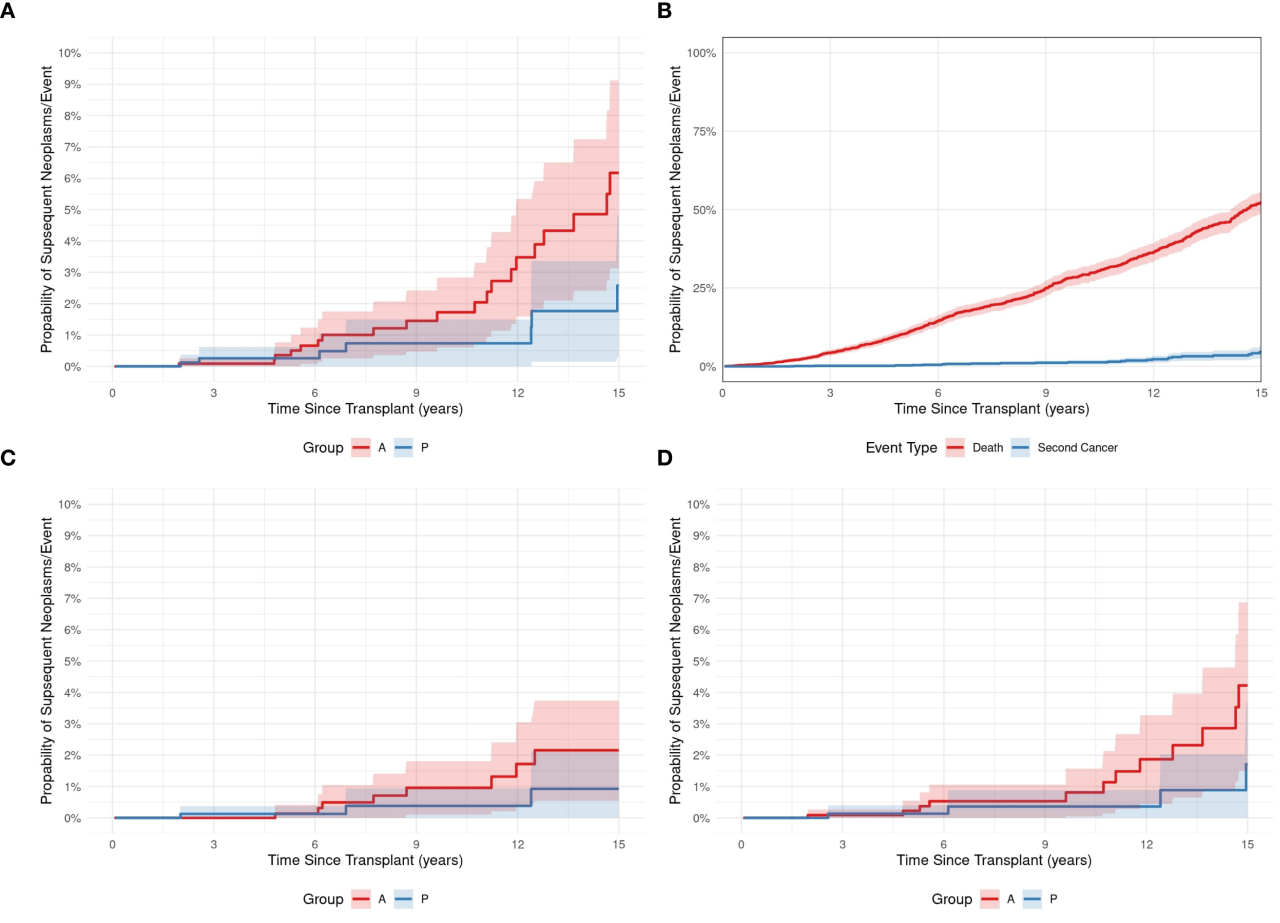
**(A)** The cumulative incidence of subsequent neoplasms in pediatric (P) and adult **(A)** HCT recipients. **(B)** The cumulative incidence of SN development (Blue plot) and death (red plot) after a subsequent neoplasm in 43 pediatric and adult HCT recipients. **(C, D)** The cumulative incidences of hematologic malignancy **(C)** or solid tumors **(D)** developing as a subsequent neoplasm after HCT in pediatric (P) and adult **(A)** patients.

**Figure 2:**
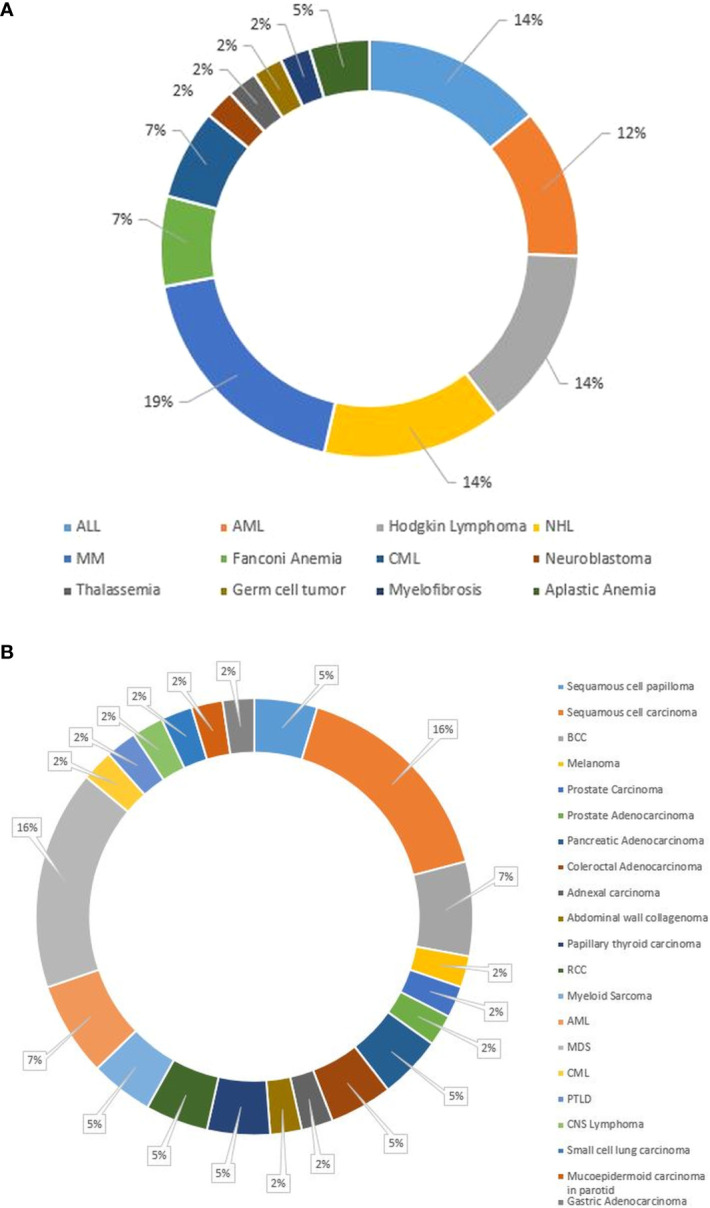
**(A)** Pie chart of primary diagnoses of the 43 pediatric and adult patients who experienced a subsequent neoplasm after HCT. **(B)** Pie chart showing the subsequent neoplasm diagnoses in our cohort of pediatric and adult HCT recipients. ALL, acute lymphoblastic leukemia; AML, acute myeloid leukemia; BCC, basal cell carcinoma; CML, chronic myeloid leukemia; CNS, central nervous system; HCT, hematopoietic stem cell transplantation; MDS, myelodysplastic syndrome; MM, multiple myeloma; NHL, non-Hodgkin lymphoma; PTLD, Post-transplant lymphoproliferative disease; RCC, renal cell carcinoma.

At a median of 8.98 years (range, 0.13–72 years) post-HCT, SNs occurred in 43 (1.6%) patients (**Table 2**). Median age at SN diagnosis was 21 years (range, 0.2–66) for the entire cohort: 8 years (1.95–17.7) for pediatric patients and 44.8 years (21.7-63) for adult patients. Twenty-eight (65%) patients were older than 18 years, and 32 (74%) were male. Twenty-three (53%) patients received allogeneic and 20 (47%) autologous HCTs, respectively. Twenty (87%) allogeneic HCTs were from full HLA-matched donors; 2 (8.6%) were from UCB, and 1 (4.4%) was from an HLA-mismatched donor. Although the difference between adult and pediatric patients’ CI of SNs (**Figure 1A**) did not reach statistical significance (p= 0.133), adult patients had a progressively increased risk over time, rising from 0.1% at 3 years to 6.2% at 15 years post-transplant. Pediatric patients demonstrated a more stable pattern, with a CI of 0.3% at both 3 and 5 years, increasing to 2.6% at 15 years. No SNs were observed in either group within the first year post-transplant. Gender-based analysis also showed no statistically significant difference in the CI of SNs between males and females (p= 0.472). By 15 years post-HCT, the CI was 5.2% (95% CI: 1.2%–9.1%) in females and 4.2% (95% CI: 1.6%–6.8%) in males. The CI of SNs for the entire cohort was 1.3%. The probability of incidence of SNs at 5, 10, and 15 years after HCT was 0.3%, 1.3%, and 4.6%, respectively (**Figure 1B**). The underlying indication for the majority (79%) of the HCTs performed at KHCC during the study period was malignancy. Of the 43 patients in whom post-HCT SNs developed, 6 (13.9%) had ALL, 5 (11.6%) had AML, 3 (7%) had CML, 1 (2.3%) had idiopathic myelofibrosis, 12 (28%) had lymphoma, 8 (18.6%) multiple myeloma, 2 (4.7%) had aplastic anemia, and 2 (4.6%) had solid tumors. In 4 (9.3%) pediatric patients, the indication for HCT was a nonmalignant condition: 3 (7%) had Fanconi anemia, and 1 (2.3%) had thalassemia (**Figure 2A**). TBI-based regimens were utilized in 14 (32.5%) patients; cGVHD occurred in 17 of 23 Allogenic HCT (74%), and cytomegalovirus infection/reactivation occurred in 26 (60.5%) patients. Sources of stem cells included PBSCs in 35 (81.4%) patients, BM in 6 (13.9%), and UCB in 2 (4.7%). MAC was administered to 35 (81.4%) patients, and RIC to 8 (18.6%). Median follow-up from the time of SN diagnosis was 10.3 years (range, 0.41–15.9 years). There was no clear temporal pattern of SN development among diseases.

**Table 2:** Characteristics of patients and features of HCTs after which a subsequent neoplasm developed.

Characteristics	Adult patients *n* (%)^*^	Pediatric patients *n* (%)^*^	N (%)
No. of patients	28 (65)	15 (35)	43 (100)
Median age at diagnosis of primary disease (years)	44.8 (21.7–63)	8 (1.95–17.7)	–
Sex			
* Male*	22 (78.6)	10 (66.7)	32 (74)
* Female*	6 (21.4)	5 (33.3)	11 (26)
No. of patients requiring HCT	28 (65)	15 (35)	43 (100)
Median age at HCT (years)	46.3 (21.9–64.8)	11.3 (2.8–24.5)	n.a.
Indication for HCT			
Malignancies	26 (93)	11 (73.3)	37 (86)
* Hematologic*	26 (93)	10 (66.7)	
* Solid Tumor*	0	1 (10)	
* Brain tumor*	0	0	
Nonmalignant conditions	2 (7)	4 (26.7)	6 (14)
Types of HCTs and Conditioning Regimens			
Allogeneic HCT	9 (31.2)	14 (92.4)	23 (53.5)
* MAC*	4	13	
* RIC*	5	1	
Autologous HCT	19 (67.8)	1 (6.6)	20 (46.5)
* MAC*	17	1	
* RIC*	2	0	
Donor type			
* Related*	9 (100)	12 (85.7)	21 (91.3)
* Unrelated*	0	2 (14.3)	2 (8.7)
HLA status of the donor			
* HLA-matched*	9 (100)	11 (78.6)	20 (87)
* HLA-mismatched*	0	3 (21.4)	3 (13)
TBI included in the conditioning regimen			
* Yes*	7 (25)	7 (46.7)	14 (32.5)
* No*	21 (75)	8 (53.3)	29 (67.5)
Follow-up after HCT			
< 10 years	21 (75)	7 (46.7)	28 (65)
*≥to 10 years*	7 (25)	8 (53.3)	15 (35)

^*^ Data are presented as the median number of patients (%), unless otherwise indicated.

HCT, hematopoietic cell transplantation; HLA, human leukocyte antigen; MAC, myeloablative conditioning; No., number; RIC, reduced-intensity conditioning; TBI, total body irradiation.

### Subsequent hematologic malignancies

Subsequent hematologic malignancies (SHMs) developed in 14 (32.5%) patients (**Figure 2B**) at a median age of 40.7 years. SHMs were mostly of myeloid origin (3 AML, 1 CML and 7 MDS) (**Figure 2B**), with a median onset time from HCT of 2.84 years (**Table 3**). Eight (57%) patients received etoposide in their conditioning regimens, and 2 (14%) received TBI. Twelve (85.7%) patients received MAC before HCT. Ten (71.4%) received an autologous HCT, and 4 (28.6%) received an allogeneic HCT.

**Table 3:** Characteristics of subsequent neoplasms that developed in pediatric and adult recipients after HCT.

Pt no.	Age at diagnosis of primary disease (y)	Primary diagnosis	Age at HCT (y)	Type of HCT	Type of donor	Conditioning regimen	Conditioning agents	GVHD	SN Diagnosis	Time from HCT to SN (y)	Treatment response of SN	Patient status	Cause of death	Time from SN diagnosis to death (months)
1	28	HL IIB	30	Auto	–	MAC	BEAM	–	AML	5.16	Non-remission	Dead	Pulmonary hemorrhage 2^nd^ to aspergillosis	1.5
2	1.95	HRNB	2.8	Auto	–	MAC	CEM	:::	AML	5.56	CR	Alive/CR	–	–
3	4	FA	6.5	Allo	UCB	MAC	FLU/CY/ATG	Acute Chronic Skin GIII, mouth	Metastatic SCC of the tongue	5.7	Metastatic	Dead	SN	11.5
4	23	T-ALL	23.6	Allo	MSD	MAC	CY/TBI	Acute gut GII	Metastatic nodular melanoma stage III	1.34	Metastatic	Dead	SN	4
5	6	CML	9.6	Allo	MSD	MAC	BU/CY	Chronic skin GI	Myeloid sarcoma	1.91	CR	Alive/CR	–	–
6	39	HL	44.7	Auto	–	MAC	BEAM	–	MDS-EBV Monosomy 7	4.65	Non-remission	Dead	Sepsis/SN	7.2
7	23	Pre-B ALL	23.9	Allo	MSD	MAC	CY/TBI	Acute Chronic mucosal/GI	SCC of the lip	2.22	CR	Alive/CR	–	–
8	63	MM	64.2	Auto	–	RIC	Melphalan	–	RCC	0.19	Non-remission	Dead	Sepsis/SN	9.5
9	50	MM	50.7	Auto	–	RIC	Melphalan	–	Myeloid sarcoma	2.76	Non-remission	Dead	Sepsis/SN	0.3
10	8	FA	8.2	Allo	MSD	MAC	CY/FLU/ATG	Chronic skin, GIII	CNS lymphoma	11.8	CR	Alive/CR	–	–
11	62	HL	64.8	Auto	–	MAC	TEAM	–	MDS Monosomy 7	0.26	CR	Alive/CR	–	–
12	7	Pre-B ALL	15.7	Allo	MSD	MAC	CY/TBI	Acute, chronic lung/mouth/eye	SCC of the maxillary sinus	13.34	Non-remission	Dead	SN	2.9
13	9	AML	9.8	Allo	MSD	MAC	CY/TBI	Chronic, mucous membranes and liver	Abdominal wall collagenoma	10.23	CR	Alive/CR	–	–
14	8	T-ALL	11.2	Allo	MSD	MAC	CY/TBI	Chronic, mucous membranes	BCC	9.69	CR	Alive/CR	–	–
15^*^	48	AML/MDS	48.8	Allo	MSD	RIC	CY/TBI	Chronic, mucous membranes	Low-risk prostate carcinoma/Schwannoma	11.48	CR	Alive/CR	–	–
16^*^	9	AML	12.5	Allo	MRD	MAC	CY/TBI	Chronic	Adnexal carcinoma with pillar differential of forehead/seborrhoic keratosis of the thigh]	5.93	CR	Alive/CR	–	–
17	8.6	Thalassemia major	11.5	Allo	MSD	MAC	BU/CY/ATG	Acute skin	Squamous papilloma of tonsil	1.69	CR	Alive/CR	–	–
18	14.4	AML	14.7	Allo	MSD	MAC	BU/CY	Chronic mouth, skin, liver	SCC of lower lip	13.38	CR	Alive/CR	–	–
19	24.3	NHL	27.6	Auto	–	MAC	BEAM	–	SCC of lower lip	0.57	CR	Alive/CR	–	–
20	23.5	HL	36.7	Allo	Haploidentical (6/10 sister)	RIC	CY/FLU/TBI/CY post	–	MDS/deletion 7	0.32	CR	Alive/CR	–	–
21	7.8	FA	8	Allo	MSD	RIC	FLU/CY/ATG	Acute skin Chronic skin	SCC	12.74	CR	Alive/CR	–	–
22	50.4	NHL	56.8	Auto	–	MAC	BEAM	–	MDS/RAEB-II	2.55	MRD	Dead	Multisystem chronic GVHD	3.6
23	17.7	HL	24.5	Auto	–	MAC	BEAM	–	CML	3.68	Non-remission	Dead	SN	–
24	45.2	NHL	46.3	Auto	–	MAC	BEAM	–	Pancreatic adenocarcinoma	2.37	Stable disease	Alive	–	–
25	53.6	NHL	55.4	Auto	–	MAC	BEAM		MDS	2.93	CR	Alive/CR	–	–
26	12.2	Mixed germ cell tumor	14.4	Allo	MRD	MAC	CY/TBI	Acute skin	AML, M4	11.77	CR	Dead	SN	7.5
27	2.4	Idiopathic myelofibrosis	16.1	Allo	MRD	MAC	BU/CY/ATG	Chronic skin, mucous membrane, lung, eyes	Squamous cell papilloma	14.67	CR	Alive/CR	–	–
28	3.6	Pre-B ALL	9.53	Allo	UCB	MAC	CY/ATG/TT/ TBI	Acute Skin stage II, GI	Papillary thyroid carcinoma	13.9	CR	Alive/CR	–	–
29	33.7	CML with myeloid blast crisis, BRC–ABL^+^	33.9	Allo	MSD	MAC	CY/TBI	Acute skin and gut/chronic skin, liver, gut, lung	Pancreatic Head adenocarcinoma	16.09	On treatment	Alive	–	–
30	42.3	MM	43.3	Auto	–	MAC	Melphalan	–	Colorectal carcinoma	8.88	Non-remission	Dead	SN	8.8
31	58.1	MM	60.6	Auto	–	MAC	Melphalan	–	Clear-cell RCC	7.9	CR	Alive/CR	–	–
32^*^	53.6	NHL	55.8	Auto	–	MAC	BEAM	–	Prostate adenocarcinoma/nasal BCC/cheek BCC	5.68	CR	Alive/CR	–	–
33	56.6	HL	57.8	Auto	–	MAC	BEAM	–	SCC of lower lip and scalp/abdominal high-grade lymphoma	6.81	Non-remission	Dead	Sepsis/SN	0.3
34	40.6	NHL	57.2	Auto	–	MAC	BEAM	–	MDS	2.23	Non-remission	Dead	Sepsis/SN	4.8
35	23.4	B ALL	23.9	Allo	MSD	MAC	CY/TBI	Chronic mouth, skin Gvhd.	Small cell lung carcinoma	12	Non-remission	Dead	SN	16
36	52.8	AML	53.3	Allo	MSD	RIC	TBI/FLU	Acute skin Gvhd Grade II, Chronic mucous membranes.	Nasal BCC/soft tissue sarcoma of arm	19.6	On treatment	Alive/CR	–	–
37	15	CML/Pre-B All	15.7	Allo	MRD	MAC	CY/TBI	Chronic mouth, skin, liver	Mucoepidermoid carcinoma in parotid	6	CR	Alive/CR	–	–
38	24.4	Aplastic Anemia	24.9	Allo	MSD	RIC	CY/ATG	Acute skin, liver Chronic skin extensive	Gastric adenocarcinoma	12.7	Metastatic	Dead	SN	16
39	51	MM	59	Auto	–	MAC	Melphalan	–	MDS	2.4	Non-remission	Dead	Sepsis/AKI	10
40	21.7	Aplastic Anemia	21.9	Allo	MSD	RIC	CY/ATG	:_	PTLD	0.13	CR	Alive/CR	:_	::
41	38.9	MM	40.5	Auto	–	MAC	Melphalan	–	Papillary thyroid carcinoma	3.4	CR	Alive/CR	–	–
42	44.8	MM	45.9	Auto	–	MAC	Melphalan	–	Adenocarcinoma of rectum	0.9	metastatic	Dead	COVID-19	14
43	49.6	MM	51.4	Auto	–	MAC	Melphalan	–	BCC of the nose	0.5	CR	Dead	ESRD	13.5

AKI, acute kidney injury; ALL, acute lymphoblastic leukemia; AML, acute myelogenous leukemia; ATG, anti–thymocyte globulin; BCC, basal cell carcinoma; BEAM, bleomycin, etoposide, Ara C, melphalan; Bu, busulfan; CEM, carboplatin, etoposide, melphalan; CML, chronic myeloid leukemia; CNS, central nervous system; CR, complete remission; CY, cyclophosphamide; EB, excess blasts; ESRD, end stage renal disease; FA, Fanconi anemia; FLU, fludarabine; GI, gastrointestinal; GVHD, graft versus host disease; HL, Hodgkin lymphoma; HRNB, high-risk neuroblastoma; HSCT, hematopoietic stem cell transplantation; MAC, myeloablative conditioning; MDS, myelodysplastic syndrome; MM, multiple myeloma; MRD, matched related donor; MSD, matched sibling donor; NHL, non-Hodgkin lymphoma; Pre-B ALL, precursor B-cell acute lymphoblastic leukemia; PTLD, Post-Transplant Lymphoproliferative Disorder; RAEB, refractory anemia with excess blasts; RCC, renal cell carcinoma; RIC, reduced-intensity conditioning; SCC, squamous-cell carcinoma; T-ALL, T-cell acute lymphoblastic leukemia; TBI, total body irradiation; TEAM, thiotepa, etoposide, cytarabine, melphalan; TT, thiotepa; UCB, unrelated cord blood.

### Subsequent solid neoplasms

Subsequent solid neoplasms (SSNs) developed in most 28 (65%) patients with SNs (**Figure 2B**), at median time to onset from HCT of 6.8 years (**Table 3**). SSNs were mainly carcinoma (85%, n=24): 7 (25%), squamous cell carcinoma (SCC); 6 (21.4%), adenocarcinoma; 3 (10.7%), Basal cell carcinoma; 4 (14.3%), carcinoma; 2 (7.1%) each renal cell carcinoma, Thyroid papillary carcinoma, and squamous papilloma; and 1 (3.6%) each, melanoma and collagenoma (**Figure 2B**).

SSNs arose in patients with a median age at the time of HCT of 23.9 years and mainly in those with leukemia [6 (14%), ALL; 5 (12%), AML; and 2 (5%), CML] or lymphoma [3 (11%) non-Hodgkin lymphoma, and 1 Hodgkin lymphoma (**Figure 2A**). Most transplants were allogeneic: 13 from matched sibling donors, 3 from matched related donors (MRDs), and 2 UCB. Twenty-two (79%) patients received MAC, 12 (43%) received TBI, and 4 (14%) received etoposide in their conditioning. Characteristics of patients with SSNs are summarized in **Table 3**.

Of the entire 43 patients who developed subsequent neoplasms, only one patient, whose primary diagnosis was aplastic anemia, developed PTLD at 0.13 years post HCT (**Table 3**).

### Outcome of SNs

**Figure 3:**
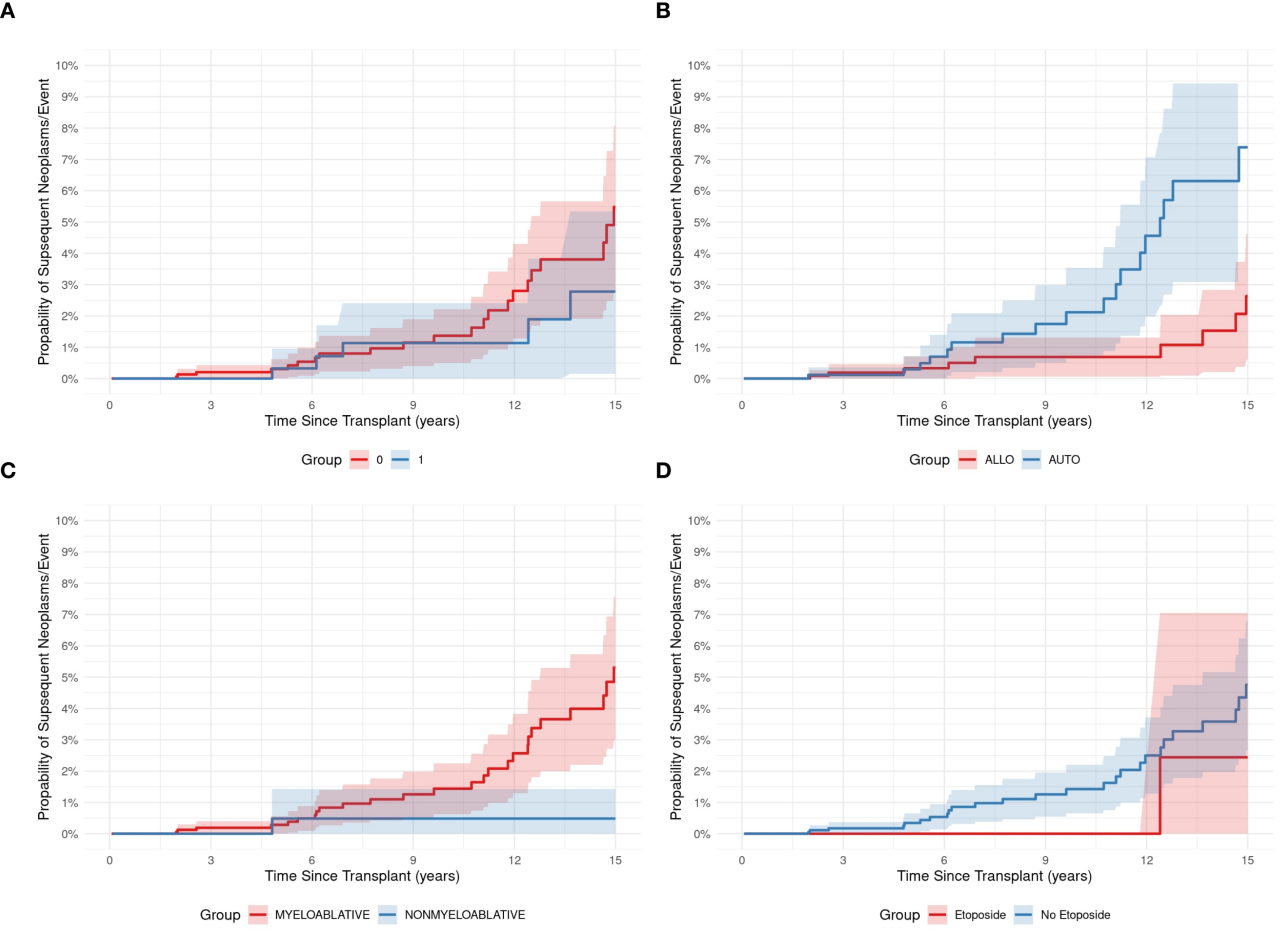
The development of subsequent neoplasms was not associated with HCT features, except for type of transplant. The use of total body irradiation (TBI; 1 *vs.* No TBI; 0) **(A)**, the type of transplant, i.e., autologous (AUTO) *vs* allogeneic (ALLO) **(B)**, the conditioning regimen (myeloablative vs reduced intensity (nonmyeloablative)) **(C)**, and the use of etoposide (Etoposide vs No Etoposide) in the conditioning regimen **(D)**.

Among the 43 patients with SNs, 25 (58%) were alive at last follow-up, and 18 (42%) had died of SNs. The 5-year OS after SN development was 58%. There was no difference in OS between patients who had received TBI *vs* no-TBI (*p* = 0.258) (**Figure 3A**), nor between patients with SHM *vs* SSNs (*p* = 0.208) (data not shown). There was also no difference in those who received RIC *vs* MAC, (*p* = 0.113) (**Figure 3C**), or with *vs.* without etoposide (*p* = 0.251) (**Figure 3D**). While the cumulative incidence of second malignancies differed significantly between patients who underwent allogeneic (Allo) and autologous (Auto) HCT. Among Allo recipients, the cumulative incidence increased from 0.0% at 1 year to 0.2% (95% CI: 0.0%-0.5%) at 3 years, 0.3% (95% CI: 0.0%-0.7%) at 5 years, 0.7% (95% CI: 0.1%-1.3%) at 10 years, and 2.6% (95% CI: 1.2%-4.0%) at 15 years post-transplant. In contrast, Auto recipients showed a steeper increase in cumulative incidence, starting at 0.0% at 1 year, reaching 0.1% (95% CI: 0.0%-0.3%) at 3 years, 0.3% (95% CI: 0.0%-0.6%) at 5 years, 2.1% (95% CI: 1.0%-3.2%) at 10 years, and peaking at 7.4% (95% CI: 5.1%-9.7%) at 15 years. (P = 0.013), indicating that patients undergoing Auto transplants had a higher cumulative incidence of second malignancies compared to those receiving Allo transplants (**Figure 3B**).

**Figure 4:**
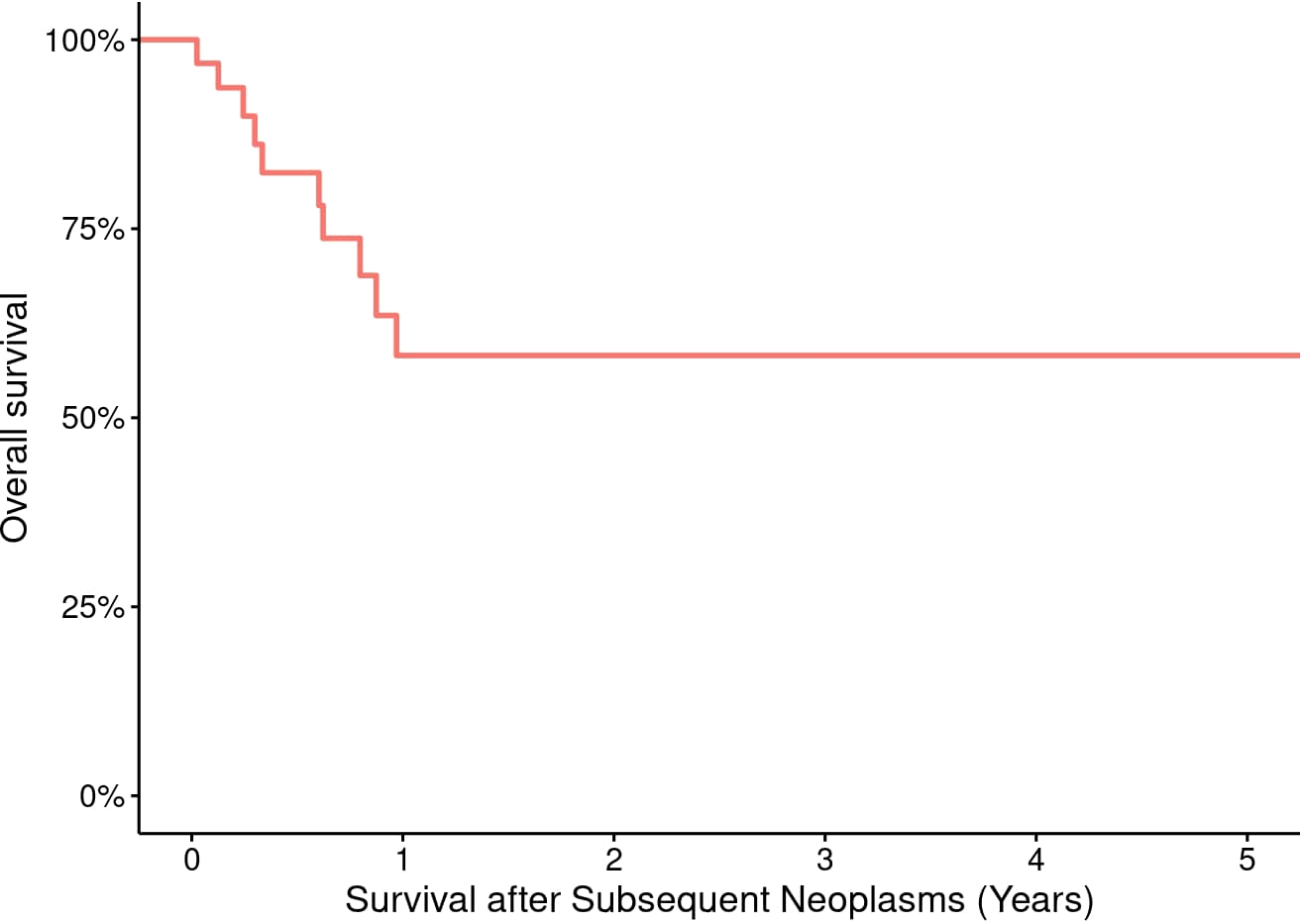
Overall survival of 43 pediatric and adult HCT recipients who experienced a subsequent neoplasm.

Survival data are presented in **Table 3**. OS probabilities after SN development at Year 1 was 58.2% (95% CI: 40.9% to 82.9%). This survival estimate remained consistent through Year 5, with an OS probability of 58.2% (95% CI: 40.9% to 82.9%) (**Figure 4**). The 5- and 10-year CIs of death due to SNs were 0.3 and 1.3, respectively (**Figure 1B**). SHM was the most reported cause of death (57%) after SN development, followed by SSN (36%) (**Figures 1C, D**).

## Discussion

Recent studies confirm that HCT recipients face a significantly higher risk of SN compared to the general population (34). In a Korean cohort of 5,177 allogeneic HCT recipients, the 10-year CI of subsequent non-hematologic malignancies was 4.23% versus 2.3% in matched controls (HR 1.73, 95% CI 1.32–2.25). In AML patients conditioned with chemotherapy-only regimens, the 10- and 15-year CI of SCC reached 4.2% and 8.1%, respectively, exceeding general population rates (35). Moreover, large registry data (n = 28,874) demonstrated cumulative SSN incidences of 1%, 2.2%, and 3.3% at 10, 15, and 20 years post-transplant (36), with cGVHD as a significant predictor. Pediatric ALL survivors who underwent HCT also exhibited a standardized incidence ratio of 2.6 for SN over 30 years as reported by Westerveld, A.S.R et al. (37) The incidence of SNs in our study was relatively lower than most values in the literature, but it was consistent with reports from Zamora-Ortiz et al. (10) Differences in methods and patient populations across these studies may have contributed to the variations.

Elevated SN risk after HCTs has been consistently observed, albeit with various incidences (18, 24, 30, 38). Hasegawa et al. (8) reported a CI of 4.2% of secondary malignancies at 10 years post-HCT, and Baker et al. (39) reported an estimated actuarial incidence of 9.9% for any post-HCT malignancy within 13 years and 22.0% by 30 years post-HCT (23), surpassing general population expectations. Whether a SN develops post-HCT is influenced by multiple factors, including chemotherapy, radiotherapy, immunologic environment post-HCT, immunosuppression, and reactivation of oncogenic viruses. In addition, recent evidence suggests that chronic inflammation and cytokine dysregulation play a central role in post-transplant tumorigenesis (40, 41). Conditioning regimens and GVHD trigger persistent activation of the NLRP3 inflammasome and increased levels of pro-inflammatory cytokines such as IL−1β, TNF−α, and IL−6, which can induce oxidative DNA damage, angiogenesis, and fibroblast activation (40, 42). These cytokines also activate key oncogenic pathways, including NF−κB and STAT3, supporting malignant transformation and immune evasion (43). Furthermore, elevated TGF−β levels in chronic GVHD promote extracellular matrix remodeling and stromal fibrosis, fostering a microenvironment conducive to neoplastic growth (44). Together, these processes, combined with impaired immune surveillance and the effects of viral reactivation, create a permissive environment for the development of SNs in HCT recipients. Young pediatric cancer survivors are particularly vulnerable. Among our patients who experienced SNs, 35% were pediatric. Furthermore, using certain chemotherapeutic agents (e.g., alkylators, topoisomerase II inhibitors) is a risk factor for SNs independent of TBI (29). Among our patients who experienced SNs, 12 had received etoposide as part of their conditioning regimen, 22 received high-dose cyclophosphamide, and 35 received MAC before HCT. Moreover, radiotherapy use and dose during conditioning are also risk factors for SNs (23). Eleven of 14 patients with SNs who had received 1200 cGy TBI for 3 days experienced carcinomas; the remaining 4 patients had AML, melanoma, collagenoma, and MDS, respectively. Rizzo et al. (33) demonstrated a 9-fold increased SN risk in younger patients who received TBI; this risk persists for decades after HCT and increases over time (13, 45). In contrast, some reports failed to identify TBI as a risk factor (10, 25, 46, 47,), indicating that the association between TBI and the development of post-HCT SNs is complex and not well understood (29).

In a study by Inamoto et al. (45), data from 31,867 patients who underwent a first HCT were analyzed for the development of SSN. Of these, 30% occurred after Auto HCT, while 70% developed following Allo HCT. Despite this difference, the OS probability of SSN did not show a statistically significant difference between the two transplant types. In contrast, within our cohort, the distribution of SN was more balanced, with 53.5% occurring post-Allo HCT and 46.5% post-Auto HCT. Notably, we observed a statistically significant higher cumulative incidence of SN following Auto HCT (p = 0.013). Several factors may contribute to this finding. First, AutoHCT recipients often receive intensive chemotherapy and/or radiotherapy prior to transplant, including alkylating agents and topoisomerase II inhibitors, both of which are established risk factors for therapy-related malignancies (14, 28, 48). Second, the absence of a graft-versus-tumor (GvT) effect in AutoHCT may reduce immune surveillance, potentially allowing pre-malignant or damaged cells to evade detection and progress to malignancy (49, 50). In contrast, the immune-mediated GvT effect in AlloHCT may play a role in reducing the risk of SNs by targeting abnormal clones. Third, AutoHCT is often used for relapsed or indolent malignancies such as lymphomas and multiple myeloma, where patients may live longer post-transplant, thus increasing the at-risk period for SN development (18). Finally, although chronic immunosuppression is more commonly associated with AlloHCT, underlying immune dysfunction in AutoHCT recipients, particularly in plasma cell disorders, may also promote carcinogenesis through impaired immune surveillance or persistent inflammatory signaling (51).

Cancer-predisposition disorders also contribute to SN development. Deeg et al. (52) described 18 SNs in 79 patients with FA at 6–11 years post-HCT. In our study, only 3 patients with FA experienced SNs, which could be attributed to eliminating radiotherapy and using RIC. Also, chronic mucosal inflammation associated with cGVHD and immunosuppression is associated with 2- to 3-fold higher rate of SNs than that in the general population of cancer survivors (14). Moreover, cGVHD appears to increase the risk of SCC (53); among our patients, SCC occurred in 7, and 5 of those also had cGVHD. Similarly, the risk of post-HCT SSN is more than twice that of the general cancer survivor population (54). The CI of SSN at 5, 10, and 15 years post-HCT is 2.2% (22), 6.55% (55), and 12.8%, respectively (3.8-fold higher than that in an age-matched control population) (22). Notably, SSNs tend to occur at younger ages than primary cancers (45) and exhibit longer latency post-HCT (16), with significant risks extending beyond 5 years post-HCT. Moreover, the incidence of SSN increases over time, without reaching a plateau (14, 29, 42, 55–57). Among SN subtypes, there is a high incidence of skin malignancies (54) and elevated risk of tumors of the oral cavity, esophagus, lung, soft tissue, and brain after busulfan-cyclophosphamide conditioning (58). In our study, SSNs comprised 65% of SNs; they manifested at a median of 6.8 years post-HCT and at a median age of 23.9 years. The most prevalent subtype was SSC. The incidence of these SSNs in our cohort exhibited a similar upward trend without plateauing. Leukemia and lymphoma were underlying indications for HCTs in these patients. Younger age at HCT is a major risk factor for SSNs; children younger than 10 years had a 33- to 36.6-fold higher risk of SSN than expected (22), which was 4.6 times higher than expected for those who were 10–29 years old at the time of HCT (12). In multivariate analysis, age older than 35 years at the time of autologous HCT and more than 36 months from diagnosis to autologous HCT were associated with greater SN risks (28). SHMs represent aggressive diseases characterized by a high incidence of adverse histologic and immune-biologic features, including therapy-related AML/MDS, a consequence of cumulative therapeutic exposure to pre-HCT alkylating agents, topoisomerase II inhibitors, radiation, and using PBSCs (48). Moreover, SHMs are more likely to be encountered after HCT (59). Median time to develop AML/MDS in our cohort was 29 months from the time of autologous HCT (range, 12–62 months). Among 14 patients with SHM, 10 had received autologous HCT and 4 had received allogeneic HCT. Two patients had received TBI, and 8 had received etoposide. Similar to results from a previous report (25), in our cohort, SHM accounted for 32.5% of SNs; all were of myeloid origin, mainly AML/MDS, which occurred at a median of 2.72 years post-HCT. This confirms shorter latency of AML/MDS post-HCT (59), occurring at a median of 1–2 years (16) and other leukemias developing relatively early after HCT (15, 57, 58).

The 5-year probability of OS after SN diagnosis depends on the SN type (53). Ehrhardt et al. (53) demonstrated that the highest risk of mortality after SNs occurred primarily within the first 5 years after SN diagnosis, as evidenced by 10- and 15-year OS estimates of 46% and 40%, respectively (53). Nevertheless, after 6 years from SN diagnosis, there was no significant increase in death risk linked to longer time between HCT and SN diagnosis, as per regression analysis. Among our HCT recipients who experienced SNs, 18 died; 13 died of their SN at a median of 7 months from SN diagnosis.

Similarly, outcomes of SSNs post-HCT show variability, with mortality rates approaching 100% within 5 years of SN diagnosis (53). Higher risks of mortality and poorer survival were noted in patients with SSNs at a younger age compared to the same type of primary cancer in the general population (45). Likewise, SHMs are linked to an unfavorable prognosis characterized by median survival of a few months (47). In our cohort, patients who experienced SHMs had high mortality rates, and those with AML/MDS died at a median of 6.8 months (range, 0–39.9 months) from the diagnosis of SHM.

Defining SNs can be challenging, and retrospective observational studies can have several limitations. Some events may be underreported due to survivors being lost to follow-up, leading to an underestimated incidence of SNs and, in turn, overestimated survival. The number of SNs in our cohort was limited and may reflect under-detection or benign tumors in patients and those lost to follow-up. Therefore, OS for individual tumor types should be cautiously interpreted. Moreover, the limited number of SNs and the heterogeneity of our cohort precluded our ability to investigate the impact of individual risk factors on OS or calculate carcinogenesis risk with reasonable accuracy. Additionally, subgroup analyses based on age and gender did not reveal any statistically significant differences in the incidence of SNs, likely due to the small event rate, further limiting the strength of comparative conclusions. Other limitations included a lack of comparison with age-matched controls to determine risk factors, the lack of detailed information on pre-transplant chemotherapy regimens, including the number of cycles and lines of prior treatment, as well as genetic testing, including cytogenetic and molecular data, such as TP53 mutation status. Given the large and heterogeneous nature of the cohort, spanning both pediatric and adult populations over 18 years, standardized data on pre-transplant therapies were not consistently available across all patients. Given these limitations, we could not estimate any associations between potential risk factors and SN development or prognosis. Despite these limitations, the outcomes of this historic cohort provide generalizable guidance for future HCT recipients with SNs. Future studies with more granular, prospective data collection may help clarify the influence of prior treatments on the risk of SNs post-HCT.

## Conclusions

The development of post-HCT SNs poses significant risks to the survival and quality of life of HCT recipients. The incidence of SNs continues to increase over time. Although the SN rate observed in our cohort was lower than that reported in the literature, it is imperative to conduct longer follow-up studies with larger cohorts to more accurately determine SN risks. With longer follow-up, more SSNs will develop. Moreover, the rising incidence of SNs underscores the importance of comprehensive, lifelong surveillance, screening programs, and preventive measures in mitigating the impact of SNs and improving the outcomes of HCT survivors.

## Data Availability

The original contributions presented in the study are included in the article/supplementary material. Further inquiries can be directed to the corresponding author.
